# Efeitos da Terapia com Anti-TNF alfa na Pressão Arterial em Pacientes com Hipertensão Resistente: Um Estudo Piloto Randomizado, Duplo-Cego e Controlado por Placebo

**DOI:** 10.36660/abc.202190703

**Published:** 2021-03-03

**Authors:** Ana Paula de Faria, Alessandra M. V. Ritter, Arthur Santa-Catharina, Débora P. Souza, Estephania P. Naseri, Manoel B. Bertolo, Mariana Rodrigues Pioli, Caio C. Carvalho, Rodrigo Modolo, Heitor Moreno

**Affiliations:** 1 Universidade Estadual de Campinas CampinasSP Brasil Universidade Estadual de Campinas, Campinas, SP - Brasil; 2 Universidade de São Paulo Ribeirão PretoSP Brasil Universidade de São Paulo, Ribeirão Preto, SP - Brasil

**Keywords:** Hipertensão, Pressão Arterial, Infliximab/uso terapêutico, Ensaio Clínico Controlado Randomizado, Inflamação

## Abstract

**Fundamento:**

A citocina fator de necrose tumoral alfa (TNF-α) é elevada na hipertensão resistente (HAR), mas os efeitos dos inibidores de TNF-α nessa população ainda são desconhecidos.

**Objetivos:**

O objetivo deste estudo foi avaliar se uma única dose de infliximabe controlada por placebo reduz a pressão arterial (PA) de forma aguda em pacientes com HAR.

**Métodos:**

Realizamos um estudo cruzado, randomizado, duplo-cego e controlado por placebo em que pacientes com HAR receberam infliximabe ou placebo. O desfecho primário foi a alteração dos níveis de PA média em relação ao basal imediatamente após a infusão, obtida por avaliação hemodinâmica não invasiva contínua, batimento a batimento. Os desfechos secundários incluíram alterações em medidas de PA central, ambulatorial e em consultório, na função endotelial, e nos biomarcadores inflamatórios após 7 dias. O nível de significância aceito foi alfa=0,05.

**Resultados:**

Foram incluídos dez portadores de HAR. O resultado do desfecho primário demonstrou uma redução aguda dos níveis de PA média (média das diferenças ± desvio padrão = -6,3 ± 7,2 mmHg, p=0,02) em relação ao basal, após o uso de infliximabe, em comparação com o placebo. Os níveis de PA diastólica (-4,9 ± 5,5 mmHg, p=0,02), mas não os níveis de PA sistólica (-9,4 ± 19,7 mmHg, p=0,16), reduziram após a infusão de infliximabe. Não foram identificadas diferenças significativas nos demais parâmetros hemodinâmicos, nem nos resultados dos desfechos secundários, com exceção dos níveis de TNF-α, que aumentaram continuamente após o uso de infliximabe. Não foram relatados eventos adversos durante o protocolo.

**Conclusões:**

Uma dose única de infliximabe reduziu os níveis de PA média e diastólica imediatamente após sua infusão, em comparação com placebo em HAR. A terapia com anti-TNF-α foi considerada segura e bem tolerada. Os resultados desse estudo prova de conceito são geradores de hipótese e precisam ser investigados em maior detalhe. (Arq Bras Cardiol. 2021; 116(3):443-451)

## Introdução

É sabido que a inflamação sistêmica de grau baixo é subjacente à fisiopatologia da hipertensão resistente (HAR) devido à falta de controle de pressão arterial (PA), juntamente com condições coexistentes, tais como obesidade, diabetes tipo 2 (DT2) e síndrome metabólica. Recentemente, nosso grupo de pesquisa explorou o papel das citocinas inflamatórias nessa população de alto risco. Um escore inflamatório alto, incluindo, entre outros, a citocina pró-inflamatória fator de necrose tumoral alfa (TNF-α), foi proposto a pacientes obesos com HAR em comparação com pacientes obesos com hipertensão controlada.^1^ Além disso, altos níveis de TNF-α foi associado a danos vasculares nesses pacientes com HAR.^2^

Apesar da indicação do uso de inibidores de TNF-α ser bem estabelecida para o tratamento de algumas doenças autoimunes, estudos experimentais e clínicos demonstraram seu uso efetivo para condições relacionadas ao sistema cardiovascular (CV), tais como a prevenção da hipertensão,^3^ e a redução de lesão de órgãos-alvo (LOA).^4^^,^^5^ O infliximabe apresentou benefícios cardiovasculares como um agente neutralizador de TNF-α capaz de reduzir os níveis de PA sistólica (PAS) e o remodelamento cardíaco em ratos espontaneamente hipertensos.^6^

Como o processo inflamatório é parte da HAR e o TNF-α está implicado em disfunções cardiovasculares, o objetivo deste estudo piloto de prova de conceito foi avaliar se uma única dose do inibidor de TNF-α, infliximabe, controlada por placebo, reduz os níveis de PA em pacientes com HAR.

## Métodos

### Desenho do estudo

Com o uso de um desenho cruzado de intervenção, randomizado, duplo-cego e controlado por placebo, foram explorados os efeitos agudos do infliximabe, um inibidor de TNF-α, e seu comparador, placebo constituído de soro fisiológico, como uma terapia adicional ao tratamento padrão para a população com HAR. Um esquema de randomização em blocos foi criado utilizando-se um código gerado por computador. Os pacientes com HAR foram designados aleatoriamente, na inclusão, a receber ou (1) infusão de placebo seguida de infusão de infliximabe depois de um período de wash-out de 40 dias, ou (2) infliximabe seguido de placebo depois de um período de wash-out de 40 dias. Um farmacêutico alocou, de forma não cega, os voluntários do estudo e manteve consigo os códigos de tratamento até o encerramento do estudo. O enfermeiro que preparou as infusões também o fez de forma não cega. Nenhum dos dois profissionais estava envolvido na coleta, análise ou interpretação dos dados. Um médico fez a inclusão de forma cega dos sujeitos no estudo. Os participantes, o médico avaliador e os pesquisadores que avaliaram os resultados permaneceram de forma cega após a designação das intervenções.

O estudo piloto foi de iniciativa do investigador, desenhado pelos investigadores e não houve apoio de nenhuma entidade comercial. Todos os autores garantiram que os dados e análises estivessem completos e acurados, e certificaram a aderência do estudo ao protocolo.

O estudo foi aprovado pelo Comitê de Ética em Pesquisa (número de aprovação 710.449, CAAE 30811214.9.0000.5404, da Faculdade de Ciências Médicas da Universidade Estadual de Campinas-FCM/UNICAMP, Brasil), e registrado no clinicaltrials.gov (NCT02743390). O estudo foi realizado de acordo com princípios éticos para pesquisas médicas em seres humanos da Associação Médica Mundial (Declaração de Helsinki), e todos os participantes assinaram o termo de consentimento livre e esclarecido antes de serem incluídos no estudo. Também foram seguidas as recomendações do documento *Consolidated Standards of Reporting Trials* (CONSORT).

### População

Pacientes com diagnóstico confirmado de HAR foram recrutados de uma população pré-selecionada do Ambulatório Especializado em Hipertensão Resistente do Hospital de Clínicas da Universidade Estadual de Campinas (UNICAMP, Campinas, Brasil). A HAR foi definida de acordo com a diretriz da *American Heart Association*.^7^ Foi realizado um diagnóstico preciso de HAR com um período de acompanhamento clínico de 6 meses para a triagem e a exclusão de causas secundárias de hipertensão [estenose de artéria renal (US-Doppler), feocromocitoma (metanefrinas urinárias e tomografia computadorizada), hiperaldosteronismo primário (relação aldosterona-renina>20 ng dl^−1^ por ng ml^−1^ h^−1^), síndrome de Cushing (cortisol e níveis de ACTH), apneia obstrutiva do sono (classificada como “alto risco” no questionário de Berlin)], e pseudorresistência (monitorização ambulatorial da pressão arterial (MAPA) e contagem de comprimidos para excluir a hipertensão do jaleco branco e a não aderência à medicação, respectivamente).

Os critérios de exclusão foram doença cardíaca isquêmica sintomática, função renal comprometida, histórico de acidente vascular, infarto do miocárdio e doenças vasculares periféricas, diabetes tipo 1, gravidez, tabagismo, doenças autoimunes, ou contraindicação ao uso de infliximabe. Pacientes não qualificados para o estudo também incluíram os que tinham testes tuberculínicos positivos ou inativos (latentes) ou com radiografia torácica póstero-anterior anormal - avaliada por radiologista e reumatologista especialista em infusão de TNF-α (EP).

### Protocolo e avaliações do estudo

O protocolo foi realizado com os participantes recebendo uma única infusão de infliximabe (Remicade®, 100 mg, Janssen-Cilag Farmacêutica Ltda.) na dose de 3 mg/Kg, que foi primeiro reconstituída com 10 mL de água estéril para injeção e, em seguida, diluída com 250 mL de cloreto de sódio 0,9% estéril para injeção, de acordo com as instruções do fabricante; ou recebendo uma única infusão de placebo que consistia 250 mL de cloreto de sódio 0,9% estéril para injeção. Os pacientes receberam as infusões intravenosas em um período de 2 horas, com uma vazão de 125 ml/h. Não foi coadministrada nenhuma outra medicação. O cruzamento para o braço de tratamento seguinte (infusão de placebo ou infliximabe) foi feito depois de um período de wash-out de 40 dias.

As avaliações do estudo incluíam 3 etapas (avaliação no basal, imediatamente após a infusão, e 7 dias após a infusão). Na visita para avaliação do basal (antes das infusões - T0) foram avaliados indicadores antropométricos, as PA de consultório, central (análise de onda de pulso - AOP) e níveis de MAPA, e dilatação mediada por fluxo (DMF). Além disso, foram coletadas amostras de sangue para determinar biomarcadores adicionais. Os registros hemodinâmicos não invasivos contínuos batimento a batimento foram avaliados por 15 minutos no basal (T0) e imediatamente após (T1) em ambas as infusões. A PA de consultório e a coleta de sangue também foram examinadas imediatamente após ambas as infusões (T1). Para avaliar a resposta de curto prazo devido à meia-vida longa do infliximabe (aproximadamente 8 dias),^8^ 7 dias após as infusões (T2), as PA de consultório, central e MAPA, DMF e a coleta de sangue foram reavaliadas (Figura suplementar).

Depois das avaliações do ensaio, foi respeitado um período de wash-out de 40 dias. Em seguida, o tratamento foi trocado (o que significa que participantes que receberam a primeira infusão de soro fisiológico, depois do período de wash-out de 40 dias, receberam infusão de infliximabe; e os participantes que receberam a primeira infusão de infliximabe, depois do período de wash-out de 40 dias, receberam infusão de soro fisiológico), e as avaliações de ensaio foram repetidas. (Figura suplementar).

Nenhum dos participantes alterou sua medicação anti-hipertensiva durante o período do estudo. Todos os procedimentos iniciaram às 08:00, e os parâmetros foram avaliados após 8 horas de jejum noturno. Depois do protocolo, os pacientes ainda permaneceram sob observação por 1 hora antes de serem liberados. Os participantes foram instruídos a relatar os efeitos colaterais comuns do infliximabe ou qualquer outro evento adverso que eles experimentassem em qualquer momento durante o estudo.

### Pressão arterial, função endotelial e avaliações bioquímicas

A PAS e a PA diastólica (PAD) de consultório foram avaliadas em 3 etapas do estudo – basal, imediatamente após a infusão, e 7 dias após a infusão (infliximabe e placebo) – por um profissional de saúde treinado, de acordo com as diretrizes europeias e brasileiras de hipertensão arterial. Utilizamos um esfigmomanômetro digital validado (HEM-907XL, OMRON Healthcare Inc., Bannockburn, IL, EUA). A PA ambulatorial foi medida em 2 etapas do estudo – basal e 7 dias após a infusão (infliximabe e placebo) – e foi realizada utilizando-se um monitor oscilométrico automático (Spacelabs90207, Spacelabs Inc, Redmon, WA). Os pacientes foram instruídos a manter suas atividades normais diárias e a registrar suas atividades de 24 horas em um diário pessoal.

A PAS e PAD, e a pressão de pulso foram avaliadas em 2 etapas do estudo – basal e 7 dias após a infusão (infliximabe e placebo) – e foram determinadas por AOP com o sistema Sphygmocor (Artcor, Sidney, Austrália).^9^ A onda de pulso foi obtida pelo método de tonometria de aplanação da artéria radial. O equipamento também fornece dados adicionais em relação à medição da rigidez arterial pelo índice de incremento (AIx), além do AIx corrigido para a frequência cardíaca de 75 bpm (AIx@75). O AIx é definido pela razão entre as ondas refletidas e de ejeção (onda de pulso que percorre as artérias carótida e femoral).

Os dados da avaliação hemodinâmica não invasiva contínua, batimento a batimento, foram avaliados em 2 etapas do estudo – basal e imediatamente após a infusão (infliximabe e placebo) – e foram obtidos utilizando-se o dispositivo Finometer® (Finapres Medical Systems; Amsterdam, Holanda) e o software Finometer® Beatscope Easy versão 02.10 (Finapres Medical Systems, Amsterdam, Holanda). Um manguito de dimensões apropriadas foi colocado no terceiro ou quarto dedo da mão esquerda e o braço foi deixado em repouso sobre uma mesa, com o paciente sentado. Os níveis de PA sistólica (PAS em mmHg), diastólica (PAD em mmHg), e média (PAM em mm), débito cardíaco (DC em l/min), e resistência vascular periférica total (RVPT em dyn.s/cm^−5^) foram registrados durante 15 minutos antes de imediatamente após o protocolo de infusões. Para as análises, foram utilizadas as seções estáveis de registros (os 10 minutos iniciais dos registros foram excluídos das análises). O dispositivo Finometer® utiliza o método de fotopletismografia, e fornece medidas hemodinâmicas confiáveis conforme mostrado anteriormente.^10^^,^^11^

A função endotelial foi avaliada em 2 etapas do estudo – basal e 7 dias após a infusão (infliximabe e placebo) – e foi determinada pelo método DMF, de acordo com as diretrizes atuais.^12^^,^^13^ Foi utilizado um transdutor vascular linear (7–12MHz, Toshiba Powervision 6000, Tóquio, Japão) sincronizado com sinal de eletrocardiograma (ECG) no protocolo. Os pacientes, em posição supina, em uma sala silenciosa e com ar-condicionado (22–24 °C), foram submetidos a oclusão da artéria braquial por cinco minutos, utilizando um esfigmomanômetro aneróide. O diâmetro da artéria braquial foi registrado antes e depois da compressão com manguito. A alteração do diâmetro da artéria braquial foi expressa como uma porcentagem de alteração relativa ao diâmetro do vaso imediatamente antes da inflação do manguito. O exame da função vascular foi realizado por um único examinador experiente, de forma cega. O coeficiente de variação intraobservador foi de 1,6%.

Foram coletadas amostras de sangue para avaliação em 3 etapas do estudo – basal, imediatamente após a infusão, e 7 dias após a infusão (infliximabe e placebo) – dos níveis plasmáticos de nitrato/nitrito, biomarcadores inflamatórios, tais como o TNF-α, interleucinas-6 (IL-6) e -10 (IL-10), adiponectina, proteína quimiotática de monócitos 1 (MCP-1), e os hormônios cortisol e aldosterona. Para as medições de nitrato e nitrito, foi coletado plasma heparinizado e imediatamente misturado com uma solução padrão de nitrito diluída a 5:1 contendo 0.8M de ferricianeto e 1% de NP-40.^14^ As amostras foram desproteinizadas com metanol (1:1) e centrifugadas a 14.000 g por 5 min. Depois disso, foram injetados 300 μl de sobrenadante na solução de tri-iodeto acidificada, que foi purgada com nitrogênio com um analisador de quimiluminescência em fase gasosa de óxido nítrico (ON) (Sievers Model 280i NO Analyzer, Boulder, CO, EUA). Biomarcadores inflamatórios e hormônios foram medidos em amostras de plasma coletada em EDTA e analisados pelo ensaio de imunoabsorção enzimática (R&D Systems, Minneapolis, MN, EUA), de acordo com as instruções do fabricante.

### Desfechos primário e secundários

O desfecho primário foi a alteração aguda (de T0 a T1) dos níveis de PA média em relação ao basal imediatamente após a infusão, obtida por avaliação hemodinâmica não invasiva contínua, batimento a batimento.

Os desfechos secundários incluíram alterações em: (1) níveis de PA determinados em consultório em todos os momentos de avaliação do protocolo, pela MAPA e PA central depois de 7 dias; (2) função endotelial após 7 dias; e (3) biomarcadores inflamatórios em todos os momentos de avaliação do protocolo, depois da infusão de infliximabe em comparação com placebo. Todos os resultados secundários foram exploratórios, mas foram considerados relevantes para essa população devido à natureza do ensaio – prova de conceito piloto.

### Análises estatísticas

Foi estimada uma amostra mínima de 10 pacientes com HAR para se detectar uma diferença clínica na PA média de 10 mmHg (desvio padrão de 10 mmHg) — entre infliximabe e placebo — com poder de 80% e erro tipo alfa de 0,05.

As variáveis contínuas foram expressas como média e desvio padrão (DP), devido à distribuição normal avaliada pelo teste Kolmogorov–Smirnov. As variáveis categóricas foram apresentadas como frequências e porcentagens. O teste t de Student pareado foi aplicado para comparar os valores de delta (distribuição normal) entre o infliximabe e o placebo nos mesmos pacientes (desenho cruzado). Foi realizado o teste ANOVA de dois fatores para medidas repetidas seguido do teste de comparação múltipla post hoc de Sidak para identificar as diferenças entre os tratamentos (infliximabe X placebo) nos valores delta dos momentos da avaliação (ou seja, T1-T0 – agudo, e T2-T0 – 7 dias).

As análises foram realizadas utilizando-se os softwares SPSS (IBM SPSS Statistics for Mac, Versão 21.0. Armonk, NY: IBM Corp. Lançado em 2012) e GraphPad Prism (versão 7.00 para Windows, GraphPad Software, La Jolla California EUA, www.graphpad.com). O nível de significância aceito foi alfa=0,05.

## Resultados

**Figura 1:**
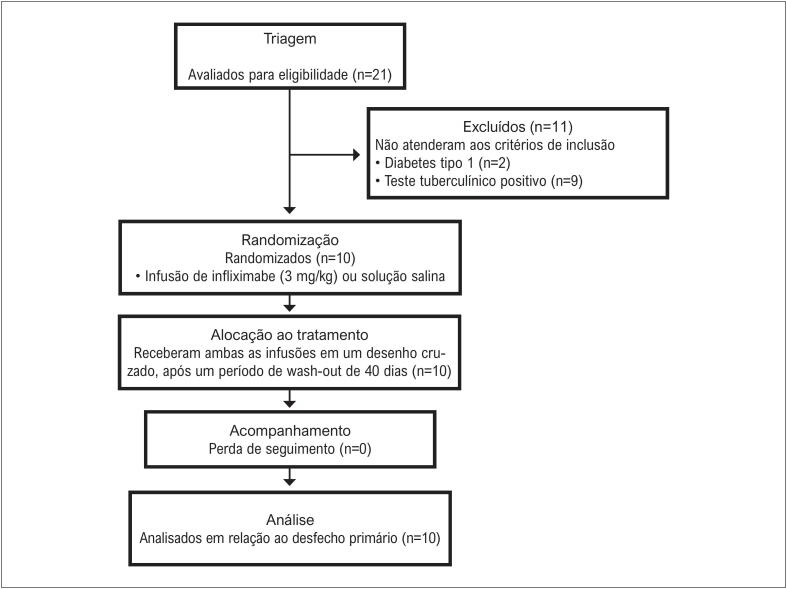
Fluxograma do estudo.

De março de 2015 a julho de 2017, foram incluídos, no total, 10 pacientes com HAR e todos os pacientes concluíram este ensaio de prova de conceito - Figura 1. As características basais dos pacientes com HAR são apresentadas na Tabela 1. A maioria dos participantes eram do sexo masculino e não brancos. Como o esperado, a maioria era obesa com DT2 e estavam tomando diuréticos e uma grande proporção de β-bloqueadores, antagonistas dos receptores de angiotensina II, e bloqueadores dos canais de cálcio.

**Tabela 1:** Características da linha de base da população do estudo

	HAR (N=10)
**Dados clínicos**	
Idade (anos)	61,8 ± 8,5
Sexo (feminino)	40% (4)
IMC (Kg/m²)	31,9 ± 5,9
Diabetes tipo 2, (n)	70% (7)
Não brancos, (n)	60% (6)
**Medicamentos anti-HA**	
Número total	4,4 ± 0,7
Diuréticos, (n)	100% (10)
Espironolactona, (n)	50% (5)
β-bloqueadores, (n)	80% (8)
IECA, (n)	30% (3)
ARA II, (n)	70% (7)
BCC, (n)	90% (9)
Agonistas alfa-2, (n)	12% (1)

Os dados foram expressos como média e desvio padrão, ou porcentagem e número absoluto. HAR: hipertensão resistente; IMC: índice de massa corporal; Anti-HA: anti-hipertensivos; IECA: inibidores da enzima conversora de angiotensina; ARA II: antagonistas dos receptores de angiotensina II; BCC: bloqueadores dos canais de cálcio.

**Figura 2:**
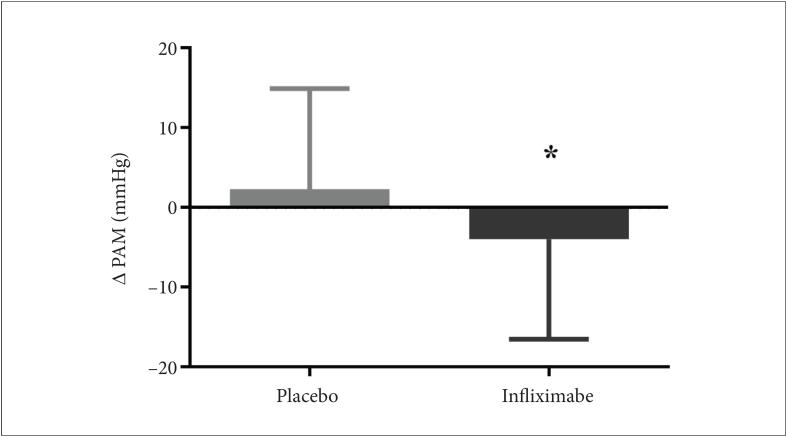
Delta absoluto da pressão arterial média (Δ PAM, 2,3 ± 12,6 versus -4,0 ± 12,5 mmHg, p=0,02) relativo ao basal imediatamente após (T1-T0) as infusões de placebo e infliximabe, respectivamente, nos pacientes estudados. Os dados foram expressos como média e desvio padrão. O teste t de Student pareado foi aplicado para comparar os valores de delta entre o infliximabe e o placebo, *p<0,05 versus placebo.

**Figura 3:**
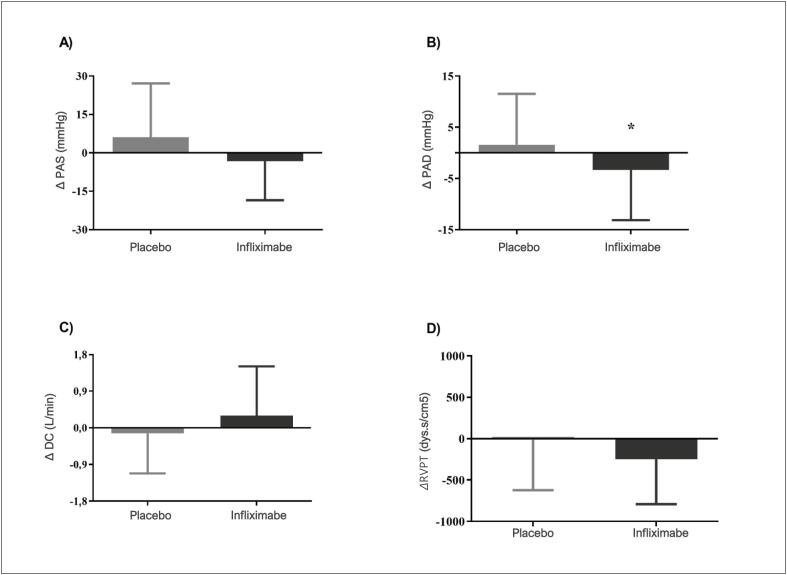
Deltas absolutos dos parâmetros hemodinâmicos batimento a batimento relativos ao basal imediatamente após (T1-T0) as infusões de placebo e infliximabe, respectivamente, nos pacientes estudados. A. Delta pressão arterial sistólica (ΔPAS, 6,1 ± 21,1 versus -3,3 ± 15,2 mmHg, p=0,16); B. Delta pressão arterial diastólica (ΔPAD, 1,6 ± 9,9 versus -3,3 ± 9,8 mmHg, p=0,02); C. Delta débito cardíaco (ΔDC, -0,14 ± 0,98 versus 0,30 ± 1,21 mmHg, p=0,44); D. Delta resistência vascular periférica total (ΔRVPT, -6,9 ± 615 versus -248 ± 543 mmHg, p=0,12). Os dados foram expressos como média e desvio padrão. O teste t de Student pareado foi aplicado para comparar os valores de delta entre o infliximabe e o placebo, *p<0,05 versus placebo.

A análise do resultado do desfecho primário demonstrou uma redução aguda dos níveis de PA média em relação ao basal, após o uso de infliximabe, em comparação com o placebo (média das diferenças ±DP foi -6,3 ±7,2 mmHg, p=0,02). Valores absolutos de delta de PA média em relação ao basal imediatamente após as infusões de placebo e infliximabe nos pacientes estudados são apresentados na Figura 2, e os demais parâmetros hemodinâmicos, na Figura 3. Além da redução dos valores de PA médios, também foi identificada uma redução dos níveis de PAD (-4,9± 5,5 mmHg, p=0,02) em relação ao basal, após o uso de infliximabe, em comparação com o placebo. Não foram identificadas diferenças estatisticamente significativas em PAS, DC e RVPT.

Os resultados dos desfechos secundários são apresentados nas Tabelas suplementares 1 e 2. Não foram identificadas alterações em PA e FC em relação aos tempos delta avaliados, T1-T0 e T2-T0, após os tratamentos. Da mesma forma, os níveis plasmáticos não apresentaram nenhuma alteração nem nos parâmetros inflamatórios nem nos hormonais, nos tempos delta avaliados, com exceção do TNF-α, que aumentou continuamente após a dose única de infliximabe, em comparação com o placebo (Tabela suplementar 1). Não foram identificadas alterações nos valores delta dos níveis de PA ambulatorial e central 7 dias após as infusões (T2-T0). A função endotelial avaliada por DMF também permaneceu inalterada (Tabela suplementar 2).

Por fim, não foram relatados pelos voluntários eventos adversos durante o protocolo das infusões, nem durante o período do ensaio. Não houve reações alérgicas ao infliximabe, e nenhum paciente abandonou o estudo devido à toxicidade.

## Discussão

O principal achado deste estudo piloto de prova de conceito foi que uma dose única de infliximabe reduziu os níveis de PA média em comparação com placebo, em pacientes com HAR. Secundariamente, o infliximabe também reduziu a PA diastólica. Até onde sabemos, o presente estudo é o primeiro a investigar os efeitos da infusão de um medicamento biológico de anticorpos monoclonais em população portadora de HAR.

Vários estudos clínicos e experimentais demonstraram o papel da citocina pró-inflamatória TNF-α na hipertensão.^3^^,^^15^ Uma relação entre o TNF-α e o sistema renina-angiotensina-aldosterona (SRAA) foi corroborada pela literatura destacando sua interação na modulação da resposta hipertensiva e da LOA a ela relacionada.^3^ Está bem estabelecido que a HAR com comorbidades existentes, tais como obesidade, DT2 e síndrome metabólica,^16^^,^^17^ apresenta hiperativação do sistema nervoso simpático (SNS) e do SRAA. Portanto, presume-se que o TNF-α seria aumentado nessa população de alto risco. Na verdade, este grupo de pesquisa já demonstrou anteriormente que os níveis de TNF-α estão aumentados em HAR em comparação com normotensos, e que essa citocina estava associada ao aumento da rigidez arterial.^2^ Recentemente, demonstrou-se também que um escore inflamatório elevado, combinando várias citocinas circulantes, tais como o TNF-α, está relacionado à HAR de maneira dependente da obesidade, em comparação com sujeitos hipertensos controlados.^1^

O uso dos inibidores de TNF-α já foi reconhecido mundialmente para tratamento de doenças autoimunes, especialmente no cenário da reumatologia.^18^^,^^19^ O infliximabe é um medicamento biológico contendo anticorpo monoclonal quimérico humano-murino que neutraliza a atividade do TNF-α. Ao realizar uma ligação com alta afinidade às formas solúveis e transmembranares do TNF-α, o infliximabe consegue inibir a ligação do TNF-α a seus receptores.^20^ Além dos benefícios clínicos observados após o uso do infliximabe para aliviar os sintomas ou evitar o avanço de doenças autoimunes, sua administração também revelou o potencial para reduzir risco cardiovascular. Por exemplo, resultados de estudos de coorte indicaram a redução na incidência de eventos CV em sujeitos com artrite reumatoide (AR) em uso de terapia anti-TNF-α.^21^^,^^22^

Os efeitos da inibição de TNF-α para prevenir o aumento dos níveis de PA e lesões a órgãos foram relatados em modelos hipertensivos.^6^^,^^23^ Este grupo de pesquisa detectou a redução na PAS e hipertrofia ventricular esquerda em ratos espontaneamente hipertensos após 8 semanas de tratamento com infliximabe (nas doses de 1,5 e 6mg/kg/semana). Esses benefícios cardiovasculares provavelmente foram alcançados devido a um mecanismo dependente de vasodilatação, em que a neutralização de TNF-α foi capaz de induzir a síntese do NO.^6^ No cenário clínico, o infliximabe (inicialmente 3mg/Kg a cada 8 semanas durante o período de monitoramento de 1 ano) diminuiu os níveis de PAS e PAD em pacientes com AR recém diagnosticada.^24^ Além disso, depois de 2 semanas de terapia, o infliximabe (3mg/kg) reduziu a PA sistólica de 24 horas em pacientes com AR, especialmente durante o período diurno. Este estudo também identificou a redução nos níveis de norepinefrina plasmática e atividade de renina plasmática, sugerindo alterações relacionadas ao infliximabe no SNS e SRAA.^25^ Os achados do presente estudo são, em parte, compatíveis com os de estudos anteriores, pois revelam um efeito modesto de uma dose única da terapia com infliximabe e de 3 mg/kg na redução dos níveis de PA imediatamente após sua infusão em uma população de risco tão alto como os portadores de HAR. Entretanto, reconhecemos a impossibilidade de atingir a meta de diferença clínica de 10 mmHg projetada neste estudo.

A inibição da via inflamatória pelo infliximabe em nosso estudo pode ter evocado de forma aguda algumas alterações funcionais/bioquímicas, e, consequentemente, acarretado esses níveis de PA reduzidos. Como bem se sabe, o TNF-α é capaz de induzir a disfunção endotelial^26^ (i) estimulando a liberação de micropartículas endoteliais e a produção de espécies reativas do oxigênio,^27^ e (ii) reduzindo a expressão de eNOS constitutiva,^28^ que reduz a biodisponibilidade de NO. Neste estudo, os resultados de DMF não apresentaram diferenças estatísticas 7 dias após a administração aguda de infliximabe, bem como os níveis de metabólitos de NO (nitrato/nitrito) em qualquer um dos dois momentos de avaliação. Embora a inibição de TNF-α tenha sido relatada como possível estratégia para melhorar a função endotelial,^29^ os resultados negativos podem ser explicados, já que foi demonstrado anteriormente que os pacientes com HAR têm vasodilatação gravemente prejudicada associada a maior rigidez vascular^30^ e a níveis mais altos de 8-isoprostano^31^ – um marcador proposto de stress oxidativo *in vivo* – comparado com hipertensos controlados. Por outro lado, ainda é possível que os níveis de NO imediatamente após o uso de infliximabe estivessem reduzidos em artérias de resistência - que este estudo não conseguiu avaliar - causando as reduções de RVPT e PA, embora esse primeiro parâmetro não tenha sido considerado significativo. Outra hipótese que corrobora nossos achados é a relação entre TNF-α e SRAA, como mencionado acima. Essa citocina pró-inflamatória pode estimular a expressão de receptores de angiotensina tipo 1,^15^ e do gene angiotensinogênio no fígado,^32^ este último levando fisiologicamente níveis altos de angiotensina II, e a secreção de aldosterona. Apenas os níveis de aldosterona foram avaliados neste estudo. Embora o resultado tenha permanecido limítrofe e o hormônio possa agir na regulação de longo prazo da PA, foi possível observar uma tendência a uma maior redução de aldosterona nos dois momentos de avaliação após o uso do infliximabe, comparado ao placebo.

É interessante que os níveis de TNF-α tenham aumentado gradualmente nos momentos da avaliação após o uso do infliximabe, em comparação com o placebo. Da mesma forma, alguns estudos detectaram o aumento em seus níveis após a terapia anti-TNF-α,^29^^,^^33^ embora os mecanismos dessa elevação ainda sejam desconhecidos. Ela possivelmente pode ser explicada pelo prolongamento da meia-vida do TNF-α pelo tratamento – conforme previamente observado nos estudos que exploram outras terapias anti-TNF-α^34^^,^^35^ – apesar de ter bloqueado a atividade do TNF-α.^34^ Por outro lado, um tratamento crônico foi associado à redução de níveis do TNF-α, atingindo um nível de equilíbrio estável mais baixo, o que pode refletir um equilíbrio entre a produção de tecido e a eliminação do TNF-α.^36^ Curiosamente, foi observada uma diminuição aguda dos níveis de PA, que não se manteve após 7 dias de acompanhamento. Como sabemos que neste estudo os níveis de TNF-α aumentaram na semana seguinte à infusão de infliximabe, e que o tratamento crônico tende a diminuir seus níveis, é razoável pressupor que um tratamento crônico poderia ser acompanhado da redução sustentada dos níveis de PA. Entretanto, isso precisa ser provado em um período de acompanhamento mais longo após a infusão.

O presente estudo tem várias limitações inerentes aos estudos piloto de prova de conceito. A limitação mais importante é o tamanho da amostra. É importante mencionar que a população estudada representa um subconjunto muito específico de hipertensos com baixa prevalência global.^16^ Além disso, tivemos uma grande proporção de pacientes com resultados positivos nos testes de tuberculose e, então, foram excluídos do estudo (n=9). Nossos achados negativos em desfechos secundários nos dois momentos de avaliação podem ter acontecido por não ter poder estatístico suficiente (erro tipo II). Uma dose única de 3 mg/kg pode não ter sido suficiente para causar o efeito clínico esperado imediatamente após a infusão ou qualquer efeito na avaliação de curto prazo (7 dias após a infusão). Embora não tenhamos alcançado a meta de diferença de 10 mmHg na PA média depois do infliximabe em comparação com o placebo [na verdade, a média das diferenças foi de -6,3 (DP=7,2)], o poder do estudo foi de 70%, e pode, portanto, ser considerado satisfatório, já que este foi um estudo piloto. Um desenho cruzado pode, reconhecidamente, implicar a possível existência de efeitos residuais da medicação. Na tentativa de superar esse problema, e considerando a meia-vida longa do infliximabe (a meia-vida de eliminação média é de cerca de 8 dias),^8^ um período de wash-out de 40 dias (5x meias-vidas) seria seguro. Como este estudo utilizou dose única, não é possível garantir que os efeitos da redução dos níveis de PA sejam sustentados durante o período de uso crônico do infliximabe. Por fim, para garantir as validades internas e externas, estudos de larga escala são necessários para estabelecer a segurança clínica e a eficácia de um inibidor de TNF-α no manejo da HAR.

## Conclusões

Este estudo piloto prova de conceito demonstrou que uma dose única de infliximabe reduziu os níveis de PA média e diastólica imediatamente após sua infusão, em comparação com placebo. A terapia com anti-TNF-α foi considerada segura e bem tolerada. Ela acrescenta uma perspectiva clínica às terapias direcionadas ao processo inflamatório para tratar da hipertensão de difícil controle. Devido ao tamanho pequeno da amostra e ao poder abaixo do pré-especificado, os achados devem ser interpretados como exploratórios e geradores de hipótese.
