# Supplementary material for: Efeitos da Terapia com Anti-TNF alfa na Pressão Arterial em Pacientes com Hipertensão Resistente: Um Estudo Piloto Randomizado, Duplo-Cego e Controlado por Placebo
Source: Arq Bras Cardiol. 2021 Mar 3;116(3):443–51. [Article in Portuguese] doi: 10.36660/abc.202190703 (PMC8159563; doi:10.36660/abc.202190703)
Supplement: Translation

# Translation: Effects of Anti-TNF alpha Therapy on Blood Pressure in Resistant Hypertensive Subjects: A Randomized, Double-Blind, Placebo-Controlled Pilot Study

**Authors:** Ana Paula de Faria, Alessandra M. V. Ritter, Arthur Santa-Catharina, Débora P. Souza, Estephania P. Naseri, Manoel B. Bertolo, Mariana Rodrigues Pioli, Caio C. Carvalho, Rodrigo Modolo, Heitor Moreno

**Affiliations:** 1 CampinasSP Brazil Universidade Estadual de Campinas, Campinas, SP - Brazil; 2 Ribeirão PretoSP Brazil Universidade de São Paulo, Ribeirão Preto, SP - Brazil

**Keywords:** Hypertension, Blood Pressure, Infliximb/therapeutic use, Randomized Controlled Trial, Inflammation

## Abstract

**Background:**

The cytokine tumor necrosis factor-alpha (TNF-α) is elevated in resistant hypertension (RH), but the effects of a TNF-α inhibitor in this population is unknown.

**Objective:**

The aim of this trial was to evaluate whether a single dose of infliximab controlled by placebo acutely reduces blood pressure (BP) in RH subjects.

**Methods:**

A double-blind, placebo-controlled, crossover trial was conducted, and randomized RH subjects received either infliximab or placebo. The primary endpoint was the change in mean BP levels relative to the baseline immediately after the infusion obtained by continuously beat-to-beat non-invasive hemodynamic assessment. Secondary endpoints included changes in office, ambulatory and central BP measurements; endothelial function; and inflammatory biomarkers after 7 days. The level of significance accepted was alpha=0.05.

**Results:**

Ten RH subjects were enrolled. The primary endpoint analysis showed an acute decrease in mean BP values (mean of differences ± standard deviation = -6.3 ± 7.2 mmHg, p=0.02) from baseline, after the application of infliximab compared with placebo. Diastolic BP levels (-4.9 ± 5.5 mmHg, p=0.02), but not systolic BP levels (-9.4 ± 19.7 mmHg, p=0.16), lowered after infliximab infusion. No further significant differences were identified in either the other hemodynamic parameters or in secondary endpoints, except for TNF-α levels, which increased continuously after infliximab infusion. No adverse events were reported during the protocol.

**Conclusions:**

A single-dose of infliximab decreased the mean and diastolic BP levels immediately after its infusion, when compared to the placebo in RH. The anti-TNF-α therapy was found to be safe and well-tolerated. The results of this proof-of-concept are hypothesis-generating and need to be further investigated. (Arq Bras Cardiol. 2021; 116(3):443-451)

## Introduction

Low-grade systemic inflammation has proven to be an underlying factor of the pathophysiology of resistant hypertension (RH) due to the lack of blood pressure (BP) control, along with coexisting conditions, such as obesity, type 2 diabetes (T2D), and metabolic syndrome. Recently, our research group has explored the role of inflammatory cytokines in this high-risk population. An elevated inflammatory score, including, among others, the proinflammatory cytokine tumor necrosis factor-alpha (TNF-α), was proposed in obese resistant subjects, when compared to controlled obese hypertensive subjects.^1^ In addition, higher levels of TNF-α have been associated with vascular damage in these RH subjects.^2^

While the recommendation for TNF-α inhibitor use is well established for treating some autoimmune diseases, experimental and clinical studies have demonstrated its effective use for cardiovascular (CV) system-related parameters, such as in preventing hypertension^3^ and reducing target organ damage (TOD).^4^^,^^5^ In fact, infliximab has revealed CV benefits as a TNF-α-neutralizing agent able to reduce systolic BP (SBP) levels and cardiac remodeling in spontaneously hypertensive rats.^6^

Since the inflammatory process is part of RH and TNF-α is implicated in CV derangements, the purpose of this proof-of-concept pilot trial was to evaluate whether a single dose of the TNF-α inhibitor, infliximab, controlled by placebo reduces BP levels in RH subjects.

## Methods

### Trial design

With the use of a randomized, double-blind, placebo-controlled interventional crossover design,this study explored the acute effects of infliximab, a TNF-α inhibitor, and its comparator placebo saline solution, as an add−on therapy to standard treatment in the population with RH. A blocked randomization scheme was created using a computer-generated code. RH subjects were randomly assigned upon enrollment to receive either (1) placebo infusion followed by infliximab infusion after a 40-day period of washout or (2) infliximab followed by placebo after a 40-day period of washout. An unblinded pharmacist distributed the study volunteers and remained with the treatment codes until study closure. The nurse who prepared the infusions was also not blinded. Both were not involved in data collection, analysis, or interpretation. A masked physician enrolled subjects into the study. Participants, evaluator physician, and the researchers assessing outcomes remained blinded after assignment to interventions.

This pilot trial was an investigator-initiated study, designed by the investigators, and no support from a commercial entity was provided. All authors certified the completeness and accuracy of the data and analyses, as well as for the adherence of the trial to the protocol.

The present study was approved by the local Research Ethic Committee (approval number 710.449, CAAE 30811214.9.0000.5404, from the School of Medical Sciences of University of Campinas-FCM/UNICAMP, Brazil) and registered in clinicaltrials.gov (NCT02743390). It was conducted in accordance with ethical principles for medical research involving human subjects established by the World Medical Association (Declaration of Helsinki), and all participants provided informed written consent before enrolling in the study. The recommendations of the Consolidated Standards of Reporting Trials (CONSORT) statement were also followed.

### Population

Subjects with confirmed diagnosis of RH were recruited from a prescreened population of the Specialized Outpatient Clinic in RH at the University of Campinas (UNICAMP, Campinas, Brazil). RH was defined according to American Heart Association Statement.^7^ A precise diagnosis of RH with a 6-month clinical follow-up was performed to screen and exclude secondary causes of hypertension [renal artery stenosis (US-Doppler), pheochromocytoma (urinary metanephrines and computed tomography), primary hyperaldosteronism (aldosterone renin ratio>20 ng dl^−1^ per ng ml^−1^ h^−1^), cushing syndrome (cortisol and ACTH levels), obstructive sleep apnea (classified as “high risk” at Berlin questionnaire)], and pseudo resistance (ambulatory BP monitoring (ABPM) and pill count to exclude white-coat hypertension and medication nonadherence, respectively).

Exclusion criteria were symptomatic ischemic heart disease, impaired renal function, liver disease, history of stroke, myocardial infarction and peripheral vascular diseases, type I diabetes, pregnant women, smoking, autoimmune diseases, or those who presented a contraindication to infliximab use. Non-eligible patients also consisted of those with a positive or inactive (latent) skin test for tuberculosis or with an abnormal posteroanterior chest radiography (chest X-ray) – evaluated by radiology and rheumatology specialists in TNF-alpha infusion (EP).

### Trial protocol and assessments

The protocol was performed with the participants, receiving either a single infliximab infusion (Remicade®, 100 mg, Janssen-Cilag Farmacêutica Ltda.) at a dose of 3 mg/Kg, which was first reconstituted with 10 mL of sterile water for injection and then diluted to 250 mL with sterile 0.9% sodium chloride for injection according to manufacturer’s instructions; or receiving a single-infusion placebo, consisting of 250 mL, with sterile 0.9% sodium chloride for injection. Subjects received the intravenous infusions over a two-hour period with a flow rate of 125 ml/h. No other drug was co-administered. Crossover to the following arm of treatment (placebo or infliximab infusion) was made after a washout period of 40 days.

Trial assessments of the study consisted of three steps (assessments in the baseline, immediately after infusion, and seven days after infusion). At the baseline visit (before infusions - T0), anthropometric, office, central (pulse wave analysis-PWA) BP, and ABPM levels and flow-mediated dilation (FMD) were assessed. Morevoer, blood samples were collected to further determine inflammatory biomarkers. Continuous beat-to-beat noninvasive hemodynamic recording were evaluated for 15 minutes in the baseline (T0) and immediately after (T1) both infusions. Office BP and blood collection were also examined immediately after both infusions (T1). To assess a short-term response due to the long half-life of infliximab (around 8 days),^8^ seven days after the infusions (T2), office, central, ABPM BP, FMD, and blood collection were revaluated (Supplementary Figure).

After the trial assessments, a 40-day period of washout was conducted. The treatment was then changed (meaning that the participant who had received saline infusion, after a 40-day period of washout, received infliximab infusion, and vice-versa; the participant who had received infliximab infusion, after a 40-day period of washout, received saline infusion), and the trial assessments were repeated (Supplementary Figure).

No participant changed their antihypertensive medication during the period of the trial. All procedures were begun at 08:00 a.m., and the parameters were evaluated after 8 hours of overnight fasting. After the protocol, patients still remained under observation for 1h before being discharged. Participants were instructed to report either common side effects of infliximab or any adverse event they could have at any time during the study.

### Blood pressure, endothelial function, and biochemical assessments

Office SBP and diastolic BP (DBP) were assessed in three steps of the study – in the baseline, immediately after infusion, and seven days after infusion (infliximab and placebo) – by a trained health professional, following the European and Brazilian guidelines of arterial hypertension. A validated digital sphygmomanometer (HEM-907XL, OMRON Healthcare Inc., Bannockburn, IL, USA) was used. Ambulatory BP measurement was assessed in two steps of the study – in the baseline and seven days after infusion (infliximab and placebo) – and performed using an automatic oscillometric monitor (Spacelabs90207, Spacelabs Inc, Redmon, WA). Patients were instructed to maintain normal daily activities and record their 24-hour activities in a personal diary.

Central systolic and diastolic aortic BP and pulse pressure were assessed in two steps of the study – in the baseline and seven days after infusion (infliximab and placebo) – and determined by PWA, using the Sphygmocor system (Artcor, Sidney, Australia).^9^ The pulse wave was obtained by the method of applanation tonometry of the radial artery. The equipment also provides additional data regarding the measurement of arterial stiffness by the augmentation index (AIx) as well as by AIx corrected for heart rate of 75 bpm (AIx@75). AIx is defined by the ratio between the reflected and the ejection waves (pulse wave traveling from carotid to femoral arteries).

Continuous beat-to-beat non-invasive hemodynamic data were assessed in two steps of the study – in the baseline and immediately after infusion (infliximab and placebo) – and were obtained using the Finometer® device (Finapres Medical Systems; Amsterdam, Netherlands) and Finometer® Beatscope Easy software, version 02.10 (Finapres Medical Systems, Amsterdam, Netherlands). An appropriately sized cuff was placed on the third or fourth left finger, and the arm was rested on a table with the subject in a sitting position. Systolic (SBP in mmHg), diastolic (DBP in mmHg), and mean (MBP in mmHg) BP levels, cardiac output (CO in l/min), and total peripheral resistance (TPR in dyn.s/cm^−5^) were recorded for 15 minutes before and immediately after the protocol of infusions. For analyses, the stable section of beat-to-beat recordings were used (10 minutes at the beginning of recordings were excluded from these analyses). The Finometer® device makes use of the volume-clamp method and provides reliable hemodynamic measures, as previously shown.^10^^,^^11^

The endothelial function was assessed in two steps of the study – in the baseline and seven days after infusion (infliximab and placebo) – and was determined by the FMD method, in accordance with current guidelines.^12^^,^^13^ Linear vascular transducer (7–12MHz, Toshiba Powervision 6000, Tokyo, Japan), synchronized with an electrocardiogram (ECG) signal, was used in the protocol. Subjects in a supine position in a quiet, air-conditioned room (22–24°C) were submitted to brachial artery occlusion for five minutes, using an aneroid sphygmomanometer. Brachial artery diameter was recorded before and after cuff compression. Change in the brachial artery diameter was expressed as a percentage change relative to the vessel diameter immediately before cuff inflation. The vascular function examination was performed by only one experienced blinded examiner. The intraobserver coefficient of variation was 1.6%.

Blood samples were collected for assessment in three steps of the study – in the baseline, immediately after infusion, and seven days after infusion (infliximab and placebo) – of the plasma levels of nitrate/nitrite; inflammatory biomarkers, such as TNF-α, interleukins-6 (IL-6), and -10 (IL-10); adiponectin; monocyte chemoattractant protein-1 (MCP-1); and the cortisol and aldosterone hormones. For nitrate and nitrite measurements, heparinized plasma was collected and immediately mixed with a nitrite-preserving solution in a 5:1 dilution, containing 0.8M ferricyanide and 1% NP-40.^14^ The samples were deproteinized with methanol (1:1) and centrifuged at 14,000g for 5 min. After, 300μl of supernatant were injected into the acidified triiodide solution and purged with nitrogen line with a gas-phase chemiluminescent nitric oxide (NO) analyzer (Sievers Model 280i NO Analyzer, Boulder, CO, USA). Inflammatory biomarkers and hormones were measured in EDTA-collected plasma samples and analyzed by enzyme-linked immunosorbent assay (R&D Systems, Minneapolis, MN, USA), according to manufacturer’s instructions.

### Primary and secondary endpoints

Our primary endpoint was the acute change (from T0 to T1) in mean BP levels relative to the baseline immediately after the infusion, obtained by continuous beat-to-beat non-invasive hemodynamic assessment.

Secondary endpoints included changes in: (1) BP levels determined by office in all timelines, ambulatory, and central measurements after seven days; (2) endothelial function after 7 days; and (3) inflammatory biomarkers in all timelines, after infliximab infusion compared with placebo. All secondary outcomes were exploratory but were considered relevant in this population due to the nature of the trial – pilot proof-of-concept.

### Statistical analyses

This study estimated a minimum sample size of 10 RH individuals, to detect a clinical difference in mean BP of 10 mmHg (standard deviation of 10 mmHg) — between infliximab and placebo — power of 80% and alpha error of 0.05.

Continuous variables were expressed as mean and standard deviation (SD), since their normal distribution was assessed by the Kolmogorov–Smirnov test. Categorical variables were presented in frequencies and percentages. Paired Student’s t-test was applied to compare delta values (normal distribution) between the application of infliximab and placebo in the same patients (crossover design). A two-way repeated measure ANOVA, followed by a Sidack’s post-hoc multiple comparison test, was performed to identify differences between the treatments (infliximab versus placebo) in the delta values of the evaluation times (i.e. T1-T0 – acute, and T2-T0 – 7 days).

The analyses were performed using the software SPSS (IBM SPSS Statistics for Mac, Version 21.0. Armonk, NY: IBM Corp. Released 2012) and GraphPad Prism (version 7.00 for Windows, GraphPad Software, La Jolla California USA, www.graphpad.com). The level of significance accepted was alpha=0.05.

## Results

From March 2015 to July 2017, a total of 10 subjects with RH were included in this study, and all subjects completed this proof-of-concept interventional trial (Figure 1). Baseline characteristics of the RH subjects are presented in Table 1. Most participants were male and non-white. As expected, they were mainly obese with T2D, and taking diuretics and a great proportion of β-blockers, angiotensin II receptor antagonists, and calcium channel blockers.

**Figure 1:** Study flow diagram.

**Table 1:** Baseline characteristics of the studied subjects

	RH (N=10)
**Clinical data**	
Age (years)	61.8 ± 8.5
Gender (Female)	40% (4)
BMI (Kg/m^2^)	31.9 ± 5.9
Type 2 Diabetes, (n)	70% (7)
Non-white, (n)	60% (6)
**Anti-HA drugs**	
Total number	4.4 ± 0.7
Diuretics, (n)	100% (10)
Spironolactone, (n)	50% (5)
β-blockers, (n)	80% (8)
ACEis, (n)	30% (3)
ARAs II, (n)	70% (7)
CCBs, (n)	90% (9)
Alpha-2 agonists, (n)	12% (1)

Data were expressed as mean and standard deviation, or percentage and absolute number. RH: resistant hypertension; BMI: body mass index; Anti-HA: antihypertensive; ACEis: angiotensin converting enzyme inhibitors; ARAs II: angiotensin II type I receptor antagonists; CCBs: calcium channel blockers.

The primary endpoint analysis showed an acute decrease in mean BP values from baseline after infliximab infusion, when compared to the placebo (the mean of differences ± SD was -6.3 ± 7.2 mmHg, p=0.02). Absolute delta mean BP values relative to baseline immediately after the placebo and infliximab infusions in the studied subjects are shown in Figure 2, while the other beat-to-beat hemodynamic parameters are shown in Figure 3. Apart from the decrease in mean BP, this study also found a reduction in diastolic BP levels (-4.9 ± 5.5 mmHg, p=0.02) from baseline, after infliximab, when compared to the placebo. No statistically significant differences were identified in SBP, CO, and TPR.

**Figure 2:** Absolute delta mean blood pressure (Δ MBP, 2.3 ± 12.6 vs. -4.0 ± 12.5 mmHg, p=0.02) relative to baseline immediately after (T1-T0) the placebo and infliximab infusions, respectively, in the studied subjects. Data were expressed as mean and standard deviation. Paired Student’s t-test was applied to compare delta values between infliximab and placebo. *p<0.05 versus placebo.

**Figure 3:** Absolute deltas of beat-to-beat hemodynamic parameters relative to baseline immediately after (T1-T0) placebo and infliximab infusions, respectively, in the studied subjects. A. Delta systolic blood pressure (Δ SBP, 6.1 ± 21.1 vs. -3.3 ± 15.2 mmHg, p=0.16); B. Delta diastolic blood pressure (Δ DBP, 1.6 ± 9.9 vs. -3.3 ± 9.8 mmHg, p=0.02); C. Delta cardiac output (Δ CO, -0.14 ± 0.98 vs. 0.30 ± 1.21 mmHg, p=0.44); D. Delta total peripheral resistance (Δ TPR, -6.9 ± 615 vs. -248 ± 543 mmHg, p=0.12). Data were expressed as mean and standard deviation. Paired Student’s t-test was applied to compare delta values between infliximab and placebo. *p<0.05 versus placebo.

Secondary endpoints are shown in Supplementary Tables 1 and 2. No changes in office BP and HR relative to assessed delta times, T1-T0 and T2-T0, after the treatments were found. Similarly, plasma levels demonstrated no alteration in either inflammatory or hormonal parameters in assessed delta times, except for TNF-α, which increased continuously after a single dose of infliximab, when compared to the placebo (Supplementary Table 1). No difference in delta values of both ambulatory and central BP levels seven days after the infusions (T2-T0). The endothelial function assessed by FMD also remained unchanged (Supplementary Table 2).

Finally, no adverse events were reported by the volunteers during the protocol of the infusions, nor throughout the trial period. There were no overt allergic reactions to infliximab, and no patient withdrew from the study because of toxicity.

## Discussion

The main finding of this proof-of-concept pilot trial was that a single dose of infliximab reduced the mean BP levels compared with placebo in subjects with RH. Secondarily, infliximab also reduced diastolic BP. To the best of our knowledge, the present study is the first to the investigate effects of the infusion of a monoclonal antibody biologic drug in the RH population.

Several experimental and clinical studies have demonstrated the role of the proinflammatory cytokine TNF-α on hypertension.^3^^,^^15^ A crosstalk between TNF-α and renin-angiotensin-aldosterone system (RAAS) has been supported by the literature highlighting their interaction in modulating a hypertensive response and TOD related to it.^3^ It is well recognized that RH with coexisting comorbidities such as obesity, T2D, and metabolic syndrome,^16^^,^^17^ presents overactivation of both the sympathetic nervous system (SNS) and RAAS. Therefore, it is presumed that TNF-α would be increased in this high-risk population. In fact, our research group has previously shown that TNF-α levels are increased in RH when compared to normotensives and that this cytokine was associated with increased arterial stiffness.^2^ Recently, our research group also demonstrated that an elevated inflammatory score, combining several circulating cytokines, such as the TNF-α, relates to RH in an obesity-dependent manner, when compared to controlled hypertensives.^1^

Inhibitors of TNF-α have been acknowledged worldwide to treat autoimmune diseases, mainly in a rheumatology setting.^18^^,^^19^ Infliximab is a chimeric human-mouse monoclonal antibody biological drug that neutralizes the biological activity of TNF-α. Binding with high affinity to the soluble and transmembrane forms of TNF-α, infliximab is able to inhibit TNF-α binding with its receptors.^20^ Besides the clinical benefits observed after infliximab use in relieving symptoms or avoiding the progression of autoimmune diseases, its administration has also revealed the potential to reduce CV risk. For instance, results from cohort studies have indicated a reduction in the incidence of CV events in subjects with rheumatoid arthritis (RA) who were taking anti-TNF-α therapy.^21^^,^^22^

TNF-α inhibition effects on preventing the rise in BP levels and organ damage have been reported in hypertensive models.^6^^,^^23^ Our research group found a reduction in SBP and left ventricular hypertrophy in spontaneously hypertensive rats after an 8-week treatment with infliximab (in both doses of 1.5 and 6mg/kg/week). These CV benefits were possibly achieved due to a vasodilation dependent-mechanism, in which the neutralization of TNF-α was able to induce the NO synthesis.^6^ In the clinical setting, infliximab (initially at 3mg/Kg every 8 weeks during a 1-year follow-up) decreased SBP and DBP levels in patients with recent-onset RA, apart from the reduction in disease activity.^24^ In addition, after 2 weeks of therapy, infliximab (3mg/kg) reduced 24-h systolic BP in patients with RA, particularly during daytime. This study also found a reduction in plasma levels of norepinephrine and plasma renin activity, suggesting the infliximab-related changes in the SNS and RAAS.^25^ Our findings are in part consistent with those previous studies, since they revealed a modest effect of a single dose of infliximab therapy at 3 mg/Kg on reducing BP levels immediately after its infusion in a high-risk population such as the RH. Nevertheless, we recognize the failure to reach the targeted clinical difference of 10 mmHg designed in this trial.

Inhibition of the inflammatory pathway by the anti-TNF-α infliximab in our study might have acutely evoked some functional/biochemical changes, and, consequently, provided these reduced BP levels. As is well-known, TNF-α is able to induce endothelial dysfunction^26^ by (i) stimulating the release of endothelial microparticles and reactive oxygen species production^27^ and (ii) downregulating the expression of constitutive eNOS,^28^ which reduces NO bioavailability. In our study, FMD results showed no statistical difference seven days after the acute administration of infliximab, nor in the levels of NO metabolites (nitrate/nitrite), in either of two assessed times. Although TNF-α inhibition has been reported as a potential strategy to improve endothelial function,^29^ our negative results might be explained by the fact that we have previously demonstrated that RH subjects have severely impaired vasodilation associated with greater vascular stiffness^30^ and higher levels of 8-isoprostane,^31^ a proposed marker of oxidative stress *in vivo*, when compared to controlled hypertensives. On the other hand, it is still possible that NO levels immediately after infliximab infusion are reduced in small resistance arteries – which our study was unable to access – causing the TPR and BP reductions, although the first parameter proved to be insignificant. Another hypothesis to support our findings is the crosstalk between TNF-α and RAAS, as already mentioned above. This proinflammatory cytokine can stimulate both the expression of the angiotensin type 1 receptors^15^ and the angiotensinogen gene in the liver,^32^ the latter physiologically leading to high levels of angiotensin II and subsequent secretion of aldosterone. Only the aldosterone levels were assessed in our study. Although the aldosterone result was borderline, and this hormone may act in a long-term regulation of BP, a trend could be observed toward a greater reduction in aldosterone in both times assessed after infliximab, when compared theto placebo.

Interestingly, TNF-α levels gradually increased in the times assessed after infliximab use, when compared to the placebo. Similarly, some studies have found an increase in their levels after anti-TNF-α therapy,^29^^,^^33^ although the mechanisms for this elevation are still unknown. This may well be explained by the prolongation of the TNF-α half-life by treatment – as previously observed in studies exploring other anti-TNF-α therapy^34^^,^^35^ – despite having blocked the TNF-α activity.^34^ On the other hand, a chronic treatment was associated with the decrease in TNF-α levels, achieving a lower steady state level, which may reflect an equilibrium between tissue production and the elimination of TNF-α.^36^ Interestingly enough an acute decrease in BP levels was observed, which was not maintained after seven days of follow-up. Since it is known that the TNF-α levels increased in the week following infliximab infusion, and that chronic treatment tends to decrease its levels, it is reasonable to suppose that a chronic treatment could be accompanied by a sustained decrease in BP levels. However, this would need to be proven in a longer follow-up after infusion.

The present study has several limitations inherent to pilot, proof-of-concept studies. The most important limitation is the small sample size. It is worth mentioning that our studied population represents a very specific subset of hypertensive patients, with a low prevalence worldwide.^16^ Moreover, a great proportion of the subjects presented positive skin tests for tuberculosis and were excluded from the study (n=9). Our negative findings in secondary outcomes in the two assessed times may have been due to an insufficient statistical power (type II error). A single dose at 3mg/Kg may not have been enough to cause the clinical effect expected immediately after the infusion, nor any effect in the short-term evaluation (seven days after infusion). Although we did not find the targeted difference of 10 mmHg in mean BP after infliximab versus placebo [in fact, the mean of differences was -6.3 (SD=7.2)], our study power was 70% and may therefore be considered satisfactory, since it is a pilot trial. We recognize that a crossover design may imply the possible existence of carry-over effects. Trying to overcome this issue, and given the long half-life of infliximab (median elimination half-life is about eight days),^8^ a washout of 40 days (5x half-life) would be safe. Due to the single dose used in this trial, it is not possible to assure that the effects in reducing BP levels are sustained during the chronic use of infliximab. Finally, to guarantee internal and external validities larger-scale trials would be required to establish the clinical safety and efficacy of a TNF-α inhibitor in the management of RH.

## Conclusions

This proof-of concept pilot trial showed that a single-dose infliximab reduced the mean and diastolic BP levels immediately after its infusion, when compared to the placebo. The anti-TNF-α therapy was found to be safe and well-tolerated. It adds clinical perspective on therapies targeting the inflammatory process to manage difficult-to-treat hypertension. Due to the small sample size and power below the pre-specified levels, the findings must be interpreted as exploratory and hypothesis-generating.
